# Anodal Transcranial Direct Current Stimulation over the Cerebellum Enhances Sadness Recognition in Parkinson’s Disease Patients: a Pilot Study

**DOI:** 10.1007/s12311-021-01295-y

**Published:** 2021-06-22

**Authors:** Fabiana Ruggiero, Michelangelo Dini, Francesca Cortese, Maurizio Vergari, Martina Nigro, Barbara Poletti, Alberto Priori, Roberta Ferrucci

**Affiliations:** 1 Neurophysiology Unit, Foundation IRCCS Ca’ Granda Ospedale Maggiore Policlinico, Milan, Italy; 2 Department of Health Science , “Aldo Ravelli” Center for Neurotechnology and Experimental Brain Therapeutics, University of Milan, Milan, Italy; 3 III Neurology Clinic, ASST Santi Paolo E Carlo, Milan, Italy; 4 Neurology Unit, San Filippo Neri Hospital, Rome, Italy; 5 Neurology Unit, Istituto Auxologico Italiano IRCCS, Milan, Italy

**Keywords:** Parkinson’s disease, tDCS, Cerebellum, Face emotion recognition

## Abstract

Emotional processing impairments, resulting in a difficulty to decode emotions from faces especially for negative emotions, are characteristic non-motor features of Parkinson’s disease (PD). There is limited evidence about the specific contribution of the cerebellum to the recognition of emotional contents in facial expressions even though patients with cerebellar dysfunction often lose this ability. In this study, we aimed to evaluate whether the recognition of facial expressions can be modulated by cerebellar transcranial direct current stimulation (tDCS) in PD patients. Nine PD patients were enrolled and received anodal and sham tDCS (2 mA, 20 min), for 5 consecutive days, in two separate cycles at intervals of at least 1 month. The facial emotion recognition task was administered at baseline (T0) and after cerebellar tDCS on day 5 (T1). Our preliminary study showed that anodal cerebellar tDCS significantly enhanced emotional recognition in response to sad facial expressions by about 16%, but left recognition of anger, happiness, and neutral facial expressions unchanged. Despite the small sample size, our preliminary results show that anodal tDCS applied for five consecutive days over the cerebellum modulates the way PD patients recognize specific facial expressions, thus suggesting that the cerebellum plays a crucial role in recognition of negative emotions and corroborating previous knowledge on the link between social cognition and the cerebellum.

## Introduction

Parkinson’s disease (PD) is a neurodegenerative disorder characterized by motor symptoms including bradykinesia, rest tremor, and rigidity, which appear in the early stages of the disease and largely depend on dopaminergic nigrostriatal denervation. However, the nigrostriatal dopaminergic degeneration involves the non-motor loops connecting the basal ganglia to areas in the frontal cortex. This causes emotional processing impairments, leading to difficulty in facial emotion recognition (FER) [1].

Despite the existence of different cultures, humans are able to recognize a specific set of basic facial expressions. This ability is one of the major communication skills present in both humans and non-human primates [2]. The capacity to infer other people’s emotional states from their faces requires two different processes: perception and emotion recognition, crucially for normal social interaction. Impairment of these processes leads to difficulties in describing bodily sensations, physiological arousal, and feelings; expressing emotions; and identifying the emotions of others from prosody and facial expression. FER allows us to interpret, discriminate, and respond to a large number of stimuli, as well as being key in interpersonal relations and in the prediction of prosocial behaviour; therefore, this impairment is associated with heightened interpersonal difficulties. Assessing the ability to understand the emotions of others is crucial to promote adaptive functioning in social interactions. Several studies investigated cognition [3] and emotion processing in PD patients, noting changes in the emotional experience, associating subjective feeling with physiological arousal, and also the impaired production and recognition of emotions resulting from different channels, faces, or voices [4, 5].

To date, there are many studies assessing abilities in recognizing facial emotions in PD, but results have been contradictory, particularly in regard to impairments of specific emotional domains [6–8]. Thus, investigating the mechanism which underlies the disruption of FER in PD is crucial for improving the quality of life of patients and their caregivers.

Different cortical and subcortical brain regions participate in the recognition of facial emotions, including the occipital-temporal cortex, amygdala, orbitofrontal cortex (OFC), basal ganglia, right parietal cortex [9], and cerebellum [10]. Some of these structures, such as the nigrostriatal system, amygdala, and insular cortex, are affected by PD-related pathology [11]. Gray and Tickle-Degnen [12] in a meta-analysis showed that individuals with PD were more impaired in terms of the recognition of negative emotions (anger, disgust, fear, and sadness) than in the recognition of relatively positive emotions (happiness and surprise) than healthy individuals.

Previous research provides evidence about the mood-congruency effects, that is, an influence of mood on emotion perception that indicates an egocentric bias when reading other’s emotional states.

Because depression is a prominent non-motor feature of PD [13], it can influence sensitivity and selective attention towards FER. In fact, altered FER has also been observed in depressed individuals. Typically, depressed patients perform poorly on some FER tasks [14, 15]. Currently, it is unknown whether FER deficits in PD affect emotional experiences and behaviour.

Several cortical-subcortical networks are involved in the recognition and discrimination of facial emotions. The cerebellum and basal ganglia are two subcortical regions in these networks. However, evidence for their specific contributions in these networks is limited. The cerebellum’s contribution to emotional processing was established by Ferrucci et al. [16], who significantly enhanced emotional recognition in response to negative facial expressions using transcranial direct current stimulation (tDCS).

Evidence suggests that different cerebral networks relay positive and negative emotions. Because the ability to appreciate positive emotions requires more sophisticated processing of individually personalized stimuli and has features similar to “higher” cortical processing, detecting pleasant features arguably relies on phylogenetically newer circuits that significantly involve the prefrontal cortex and cortical executive system [17]. Negative emotions, such as sadness and anger, are crucial for survival and help to prepare an organism for rapid defence, which is part of a defence system designed to protect the organism from threats against the acquisition of valuable resources. Therefore, these emotions activate phylogenetically older circuits involving the cerebellum [10].

Transcranial electric stimulation of the brain is a novel and highly promising technique currently employed in both research [18, 19] and clinical practice [20–22]. Improving or rehabilitating brain functions by modulating excitability using this non-invasive technique is an exciting new area in neuroscience [23, 24]. Since there are connections between the cerebellum and cerebral regions involved in motor, associative, and affective functions, the cerebello-thalamo-cortical pathway is an interesting target for this new technique.

The aim of this study was to investigate the role of cerebellum in processing emotional information in PD patients using tDCS as a novel way of modulating the excitability of remote cortical regions and their functions.

## Materials and Methods

### Participants

Nine patients aged 42–77 years, including four women (Hoehn & Yahr scale score 2–3; Mini Mental State Examination score 2–30) diagnosed with idiopathic PD, were recruited from the Fondazione IRCCS Ca’ Granda, Policlinico Hospital of Milan, Italy, and the III Neurological Clinic of the San Paolo Hospital of Milan. Patients were excluded if they had other neuropsychiatric diseases, were undergoing deep brain stimulation, or had dementia. Although depressive symptoms were not formally assessed, preliminary clinical interview excluded depressive disorders and cerebellar cognitive affective syndrome.

Throughout the tDCS study, patients continued taking their medications at the doses recommended during the previous 2 months. Demographic and clinical data for each participant are reported in Table 1.

**Table 1:** Demographic and clinical data for each participant

Patient	Gender	Age	Education (years)	MoCA score	Hohen and Yahr score	Disease duration (months)	Medications
1	Male	58	8	30	2	23	Entecapone Melevodopa + carbidopa Pramipexole Safinamide
2	Male	66	8	25	2	10	L-Dopa + benserazide L-Dopa + carbidopa Safinamide
3	Male	61	13	27	2	16	L-Dopa + benserazide Melevodopa + carbidopa Safinamide
4	Male	68	8	27	2	17	Entecapone L-Dopa + benserazide Rasagiline
5	Female	74	18	24	2.5	12	Entecapone Pramipexole Rasagiline
6	Female	42	8	26	2	6	Melevodopa + carbidopa Pramipexole Selegiline
7	Female	69	13	28	1	n.a	L-Dopa + benserazide Pramipexole Rasagiline
8	Male	66	18	27	1	n.a	L-Dopa + benserazide Rasagiline Rotigotine
9	Female	77	8	24	3	8	L-Dopa + benserazide

*MoCA* Montreal cognitive assessment

### Study Design

**Fig. 1:**
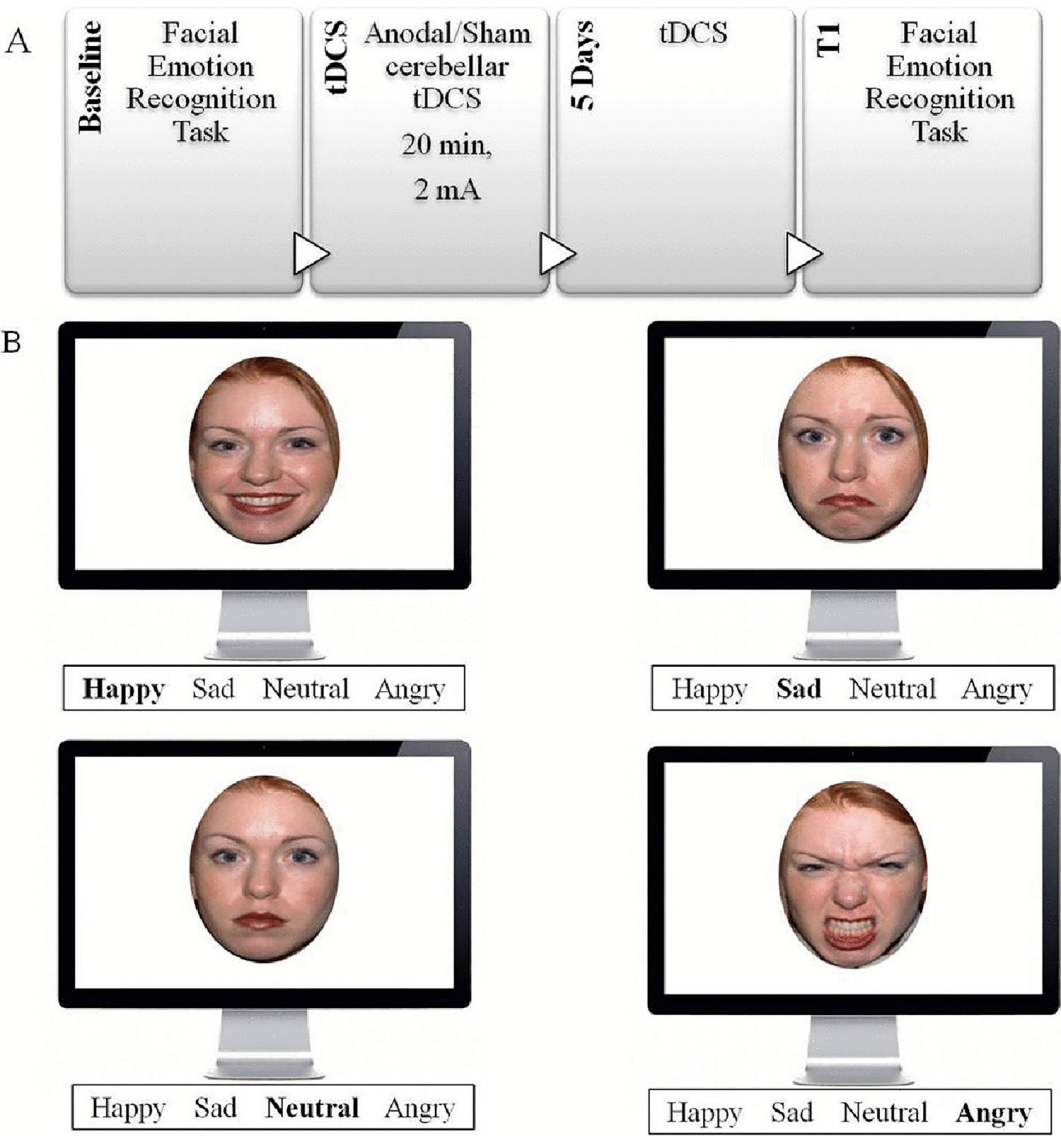
Timeline of the experimental procedure (**A**) and task stimuli (**B**). SRT, simple reaction times; VAS, visual analogue scale

This is a pilot, double blinded, sham-controlled study. All participants received anodal and sham tDCS in a random order (n = 5 started with the anodal cerebellar tDCS and n = 4 with the sham tDCS), in two independent experimental sessions separated by at least a 1-month interval. In each session, the FER task, visual analogue scale (VAS), and simple reaction time (SRT) were administered before treatment (T0) and at the end of treatment on day 5 (T1) (i.e. “offline”) (Fig. 1a). During the stimulation, participants were comfortably seated and were free to interact with the technical staff.

### Facial Emotion Recognition Task

We used the FER task proposed by Ferrucci et al. [16] (Fig. 1b). The task, extracted from the NimStim Face Stimulus Set, consisted of 16 Caucasian adults (eight men and eight women) expressing anger, happiness, sadness, and neutral expression. We generated two alternative sets of pictures consisting of 32 trials (eight faces; four men and four women). The pictures were presented in a random and balanced order, and each facial expression was shown three times, leading to a total of 96 trials (24 for each emotion category). Stimulus presentation, timing, and data collection were controlled by the E-prime (Psychology Software Tools, Pittsburg, PA) software, which was running on a laptop computer. Subjects were required to observe the faces in the pictures and assign an emotion (happiness, sadness, anger, or neutral) to each of the faces by pressing the appropriate button on a keypad as quickly and accurately as possible. Reaction times (RTs) and error rate (number of incorrect responses) were recorded.

### Simple Reaction Times

Besides the target task, we included an SRT task [25]. The SRT task was used as a control task to evaluate the presence of unspecific effects of stimulation on motor effects in the dominant hand and at the level of general attention. In this task, participants were required to press a button with their right hand as quickly as possible, when a single stimulus (a “white square”) appeared in the centre of a computer screen with delays differing from the previous response (delay range: 3000–7000 ms). This task, which consisted of 35 trials, lasted approximately 5 min.

### Visual Analogue Scale

In order to evaluate the presence of unspecific effects of general arousal that can alter emotion recognition process, a VAS measuring mood was administered. Before the FER task, the subjects completed a VAS comprising self-evaluation scale ranging from 0 to 10 consisted of a horizontal line, 100 mm in length (0 mm represented the worst mood, and 100 mm represented the best mood ever), anchored at each end by word descriptors. The subject marked the point on the line where they felt best represented how they perceived their current state. The VAS score was calculated by measuring in millimetres the distance from the left end of the line to the point that the patient marked.

### tDCS Protocol

tDCS was delivered with an electrical constant direct current stimulator (HDCKit, Newronika, Italy) connected to a pair of a rectangular saline-soaked synthetic sponge electrodes (5 × 7 cm). The stimulating current was an anodal direct current (DC) applied at 2 mA intensity (impedance < 2 kΩ) and delivered for 20 min (including 20 s at the beginning and 1 min at the end of treatment in which current was ramped up and down, respectively) in the active stimulation conditions, once a day, for five consecutive days. We used the cerebellar tDCS electrode montage described in a previous study by Ferrucci et al. [16, 26]. The stimulating electrode was placed on the median line over the whole cerebellum (1–2 cm below the inion with its lateral borders about 1 cm medially to the mastoid apophysis) and the other (return electrode) over the right shoulders. For the placebo DC, electrodes were placed similar to that for real cerebellar tDCS, but the stimulator was turned off after 10 s. Subjects therefore felt the initial itching sensation when stimulation began, but, thereafter, the current was attenuated.

### Statistical Analysis

Given the small sample size, we used a non-parametric approach. All tests were two-sided, and significance level was set as α = 0.05. Given the exploratory nature of the study, we opted not to apply a Bonferroni correction for the experiment-wise error rate, in order to reduce the risk of a type II error, as advised by [27].

We used a Wilcoxon signed-ranks test to evaluate differences at T0 in FER-RTs and FER-error rate (FER-error rate = number of errors), for each emotion between stimulation conditions (anodal vs. sham). In order to account for baseline differences in mood state and for other unspecific effects of stimulation on motor function, we analysed differences in VAS mood scores and SRTs between stimulation conditions at T0.

To evaluate polarity-specific effects, we calculated percentage (T0, 100%) FER-RTs changes after tDCS as (T1 score − T0 score)/T0 score, as well as FER-error rate change (FER-error rate change = T1 errors − T0 errors; negative values indicate a decrease in the number of errors, and vice versa). We then compared percentage changes between stimulation conditions using a Wilcoxon signed-ranks test.

In order to account for a potential role of indirect effects of tDCS on FER (i.e. owing to changes in motor speed or mood), we also analysed percentage changes in SRTs [SRT score change = (T1 score − T0 score)/T0 score] and difference in VAS mood scores (VAS mood change = T1 score − T0 score) for both stimulation conditions, using a Wilcoxon signed-ranks test.

## Results

None of the participants experienced adverse effects as a result of tDCS, and no participant withdrew from the study.

Because our objective was to evaluate whether tDCS influences FER processes, presuming that the recognition of different emotions has been proposed to depend upon the differential activation of distinct [28–32] but partially overlapping [33] brain circuits, we analysed the effects of tDCS on the recognition of four different facial expressions: sadness, anger, happiness, and neutral.

The scores of participants for the FER task are reported in Table 2. We found no significant differences for FER-RTs at T0, while we found differences for neutral facial expressions FER-error rate, which was higher in the anodal condition [median errors (IQR), anodal vs. sham = 1.00 (3.00) vs. 0.00 (1.00); Z = 2.19, *p* = 0.028]. FER-RTs at T0 were descriptively faster for happiness and anger, compared to sadness and neutral facial expressions. FER-error rate was also lower for anger and happiness, with most subjects performing at ceiling. FER-RTs and FER-error rate differences at T0 are displayed in Table 3.

**Table 2:** FER task results for the nine participants treated with anodal and sham cerebellar tDCS

	Condition	Emotion	T0 (mean ± SD)	T1 (mean ± SD)
FER-RTs	Anodal tDCS	Happiness	1371.76 ± 262.49	1380.59 ± 347.12
		Sadness	2060.06 ± 1058.54	1594.68 ± 363.66
		Anger	1397.47 ± 383.81	1346.61 ± 493.34
		Neutral	1540.15 ± 265.01	1370.24 ± 290.60
	Sham tDCS	Happiness	1503.10 ± 713.56	1393.46 ± 490.82
		Sadness	1874.06 ± 810.15	1774.49 ± 712.73
		Anger	1524.59 ± 699.75	1283.89 ± 435.22
		Neutral	1518.29 ± 468.39	1418.31 ± 420.14
FER-error rate	Anodal tDCS	Happiness	0.56 ± 1.01	1.22 ± 1.64
		Sadness	6.22 ± 6.36	3.11 ± 4.91
		Anger	1.11 ± 1.96	1.44 ± 1.94
		Neutral	1.89 ± 2.09	1.44 ± 1.33
	Sham tDCS	Happiness	1.00 ± 1.32	0.44 ± 1.01
		Sadness	5.11 ± 4.91	3.00 ± 2.50
		Anger	1.22 ± 2.95	0.89 ± 2.03
		Neutral	1.33 ± 2.18	1.33 ± 2.29
SRT	Anodal tDCS		381.63 ± 46.89	355.53 ± 57.35
	Sham tDCS		397.25 ± 167.10	408.47 ± 141.22
VAS mood	Anodal tDCS		7.89 ± 1.39	7.89 ± 1.08
	Sham tDCS		6.72 ± 2.18	7.33 ± 1.44

*FER-RTs* facial emotion recognition reaction times; *FER-error rate* facial emotion recognition error rate (number of errors); *SRT* simple reaction time; *VAS* visual analogue scale

**Table 3:** Differences in FER-RTs and FER-error rate scores at T0 between anodal and sham tDCS

	Emotion	Anodal tDCS median (IQR)	Sham tDCS median (IQR)	Z value	*p* value
FER-RTs	Happiness	1371.50 (429.24)	1124.96 (686.40)	0.18	0.859
	Sadness	1638.52 (381.23)	1476.90 (781.14)	0.88	0.374
	Anger	1287.62 (309.93)	1219.33 (483.17)	0.88	0.374
	Neutral	1496.22 (356.02)	1295.35 (364.63)	0.65	0.515
FER-error rate	Happiness	0.00 (1.00)	1.00 (1.00)	1.48	0.139
	Sadness	6.00 (8.00)	4.00 (8.00)	0.95	0.343
	Anger	0.00 (1.00)	0.00 (1.00)	0.30	0.767
	Neutral	1.00 (3.00)	0.00 (1.00)	2.19	**0.028**
SRT		387.86 (52.94)	335.80 (68.51)	1.60	0.110
VAS mood		8.00 (2.00)	7.50 (3.50)	1.66	0.097

*FER-RTs* facial emotion recognition reaction times; *FER-error rate* facial emotion recognition error rate (number of errors); *SRT* simple reaction time; *VAS* visual analogue scale. In bold: statistically significant (*p* < 0.05) differences

**Fig. 2:**
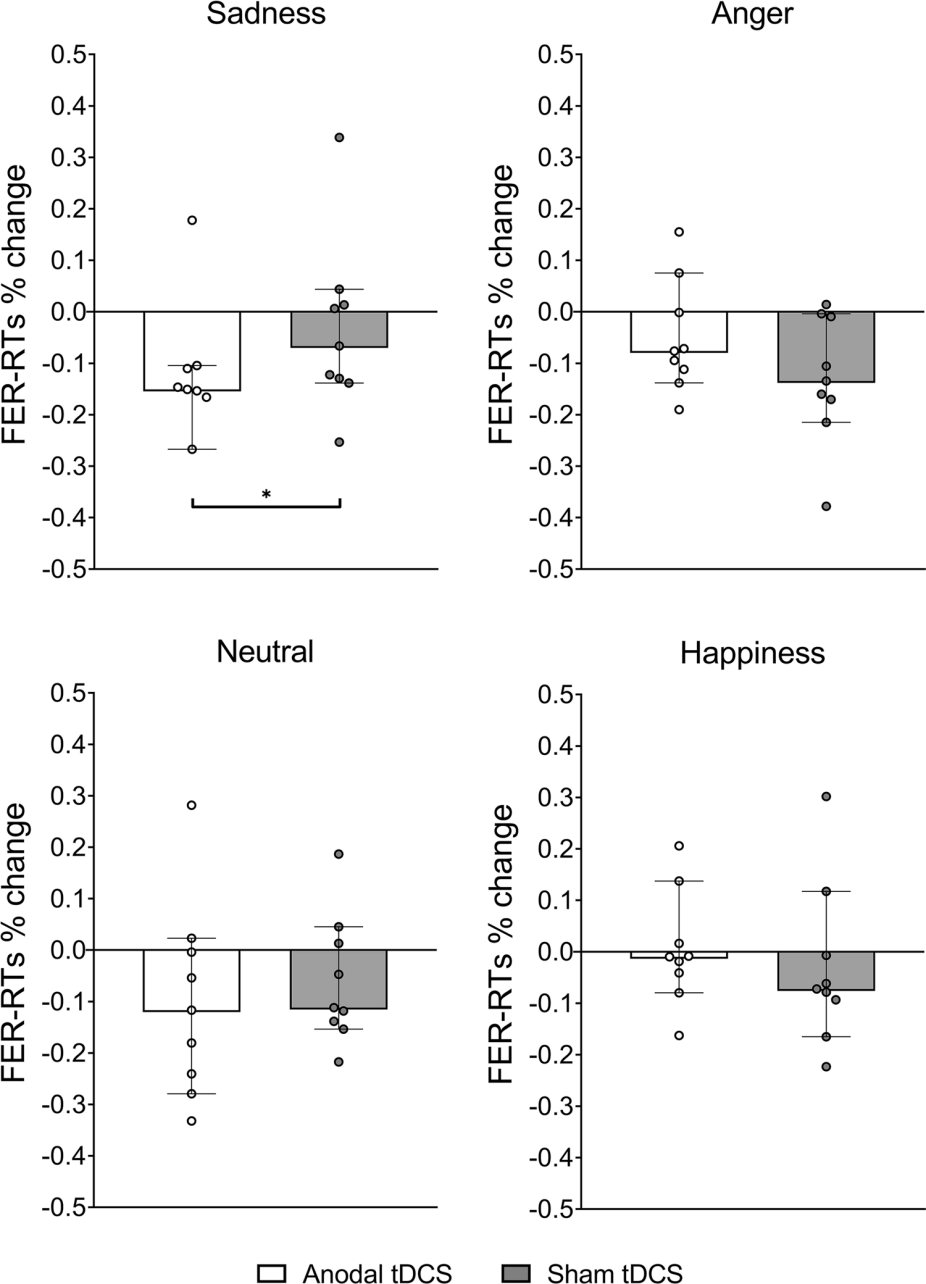
The effects of cerebellar tDCS and sham on FER-RTs % change for each emotion (sadness, anger, neutral, happiness) during the FER task. Anodal cerebellar tDCS decreased FER-RTs for sadness (*p* = 0.038). Data are displayed as median (bar height) with 95% CI (whiskers); dots represent individual FER-RTs % change between T1 and T0. Asterisks denote statistically significant differences between sham and anodal tDCS (* = *p* ≤ .05)

When we tested the effect of cerebellar tDCS on FER-RTs for each emotion, we found that FER-RTs decreased significantly only for sadness [median (IQR), anodal vs sham =  − 15.1% (5.6) vs − 6.6% (14.3); Z = 2.07, *p* = 0.038][34]. FER-RTs did not change for happiness [median (IQR), anodal vs sham =  − 0.9% (5.7) vs − 7.2% (8.6); Z = 0.77, *p* = 0.441], anger [median (IQR), anodal vs sham =  − 7.6% (11.0) vs − 13.4% (16.1); Z = 1.48, *p* = 0.139], and neutral facial expressions [median (IQR), anodal vs sham =  − 11.7% (16.1) vs − 11.2% (15.2); Z = 1.01, *p* = 0.314] (Fig. 2).

We found that FER-error rate increased for happiness only [median (IQR), anodal vs. sham = 1.00 (1.00) vs. 0.00 (1.00); Z = 2.43, *p* = 0.015). Although statistically significant, this observation might be the result of differences in FER-error rate for happiness observed between stimulation conditions at T0 [median FER-error rate (IQR), anodal vs. sham: 0.00 (1.00) vs. 1.00 (1.00); Z = 1.48; *p* = 0.139], rather than an actual effect of anodal tDCS stimulation. We did not observe statistically significant differences in FER-error rate change for negative emotions or neutral facial expressions (Table 4).

**Table 4:** Differences in FER-RTS % change and FER-error rate change between anodal and sham tDCS

	Emotion	Anodal tDCS median (IQR)	Sham tDCS median (IQR)	Z value	*p* value
FER-RTs % change [(T1–T0)/T0]	Happiness	– 0.9% (5.7)	7.2% (8.6)	0.77	0.441
	Sadness	– 15.1% (5.6)	– 6.6% (14.3)	**2.07**	**0.038**
	Anger	– 7.6% (11.0)	– 13.4% (14.2)	1.48	0.139
	Neutral	– 11.7% (23.7)	– 11.2% (15.2)	1.01	0.314
FER-error rate change (T1–T0)	Happiness	1.00 (1.00)	0.00 (1.00)	**2.43**	**0.015**
	Sadness	– 4.00 (3.00)	– 2.00 (6.00)	0.53	0.594
	Anger	0.00 (3.00)	0.00 (1.00)	0.06	0.953
	Neutral	– 1.00 (5.00)	0.00 (1.00)	0.00	1.000
SRT % change [(T1–T0)/T0]		– 8.00% (16.00)	0.01% (18.00)	1.60	0.110
VAS mood change (T1–T0)		0.00 (1.00)	0.00 (2.00)	0.47	0.635

*FER-RTs* facial emotion recognition reaction times; *FER-error rate* facial emotion recognition error rate (number of errors); *SRT* simple reaction time; *VAS* visual analogue scale. In bold: statistically significant (*p* < 0.05) differences

We did not find statistically significant differences in VAS mood scores [median (IQR), anodal vs. sham = 8.00 (2.00) vs. 7.50 (3.50); Z = 1.66; *p* = 0.097] and SRT scores [median (IQR), anodal vs. sham = 387.86 (52.94) vs. 335.80 (68.51); Z = 1.60; *p* = 0.110] between stimulation conditions at T0.

We found no differences in VAS mood score change [median (IQR), anodal vs. sham = 0.00 (1.00) vs. 0.00 (2.00); Z = 0.47; *p* = 0.635] or SRT score change [median (IQR), anodal vs. sham =  − 8.5% (16.3) vs. 0.5% (18%); Z = 1.60; *p* = 0.110] based on stimulation condition.

## Discussion

This study compared the effects of active to sham tDCS on measures of facial emotion recognition in PD patients. Our preliminary findings provide an initial proof-of-concept for the use of emotional treatment interventions paired with cerebellar tDCS for the improvement of emotion-based recognition.

In the current study, our results showed that tDCS could be an effective and reliable tool for modulating brain activity. We found that anodal cerebellar tDCS selectively modulated the perception of sadness in facial expressions. To the best of our knowledge, this is the first evidence of a relationship between cerebellar tDCS and emotional face detection in PD patients.

This study, by showing that cerebellar tDCS affected the ability to recognize sad facial expressions, extends current knowledge on the important role of the cerebellum in emotional information processing.

In fact, while the involvement of the cerebellum in motor task execution and emotion processing is well known, little is known about its mechanism [35, 36]. Schmahmann and Sherman [37] reported that patients with cerebellar lesions showed affective blunting, disinhibition, and lability, with little cognitive or behavioural change. From a neurofunctional point of view, the posterior lobes of the cerebellum are involved in both cognitive and emotion processing, particularly lobule VI, vermal lobule VII, which is part of the cerebellar-limbic circuit, and Crus I [10].

Neural changes in numerous areas and impaired dopamine transmission in the mesocorticolimbic pathway were investigated to explain deficits in FER in PD. Indeed, not only putaminal but also orbitofrontal and amygdalar presynaptic dopaminergic functions were altered during the early stages of PD [38]. Indeed, cognitively intact PD patients exhibit facial emotion recognition deficits for all basic emotions excluding happiness, when compared to healthy controls [6].

In light of cerebellar engagement in emotional information processing, the specific modulation for the recognition of negative stimuli could be ascribed to the reciprocal connections with the amygdala [16, 39]. FER deficits in PD could also be attributed to neural synchronization within the basal ganglia [5]. Studies suggest that the basal ganglia recruit and synchronize the activities of the face fusiform area, amygdala, and OFC [40, 41]. A dysfunction within basal ganglia-based circuits may, therefore, introduce noise into the system, disrupt the synchronization process, and lead to biased emotional judgements characterized by weaker emotion discrimination that could be assessed by rating tasks. The cerebellar tDCS-induced changes associated with sadness recognition that we observed in our pilot study are consistent with the findings of previous studies that showed that different neuronal circuits exist, and at least those associated with negative emotion recognition seem to involve the cerebellum. Studies have shown that specific regions of the cerebellum are activated when processing sad emotional stimuli; specifically, an fMRI study by [42] found sadness to correlate with activity in the left paravermal lobule VI and in the vermal lobule VIIIA.

Our preliminary results observed in PD patients are also in line with the findings of Park et al. [43], who found a different neural network based on the salient stimulus: the positive emotional stimulus “happiness” activates the middle temporal gyrus, parahippocampal gyrus, hippocampus, claustrum, inferior parietal lobule, cuneus, middle frontal gyrus, inferior frontal gyrus, and anterior cingulate gyrus, whereas a negative emotional stimulus activates the posterior cingulate, fusiform gyrus, and cerebellum. Consistently, impaired recognition of sadness, but not of happiness, has been observed by a recent study on patients with cerebellar lesions [44], and enhanced emotional recognition for sadness and anger (but not for happiness) has been observed following tDCS stimulation of the cerebellum in healthy subjects [16].

These findings suggest a possible involvement of the cerebellum as a part of a widespread network that enhances affect regulation and behaviour towards emotionally relevant stimuli, especially those with some negative valence such as sadness. This suggests that negative events generally evoke stronger cognitive, emotional, and social responses than neutral or positive events [45].

Behavioural data suggest that human facial expressions communicate both the emotional state of the poser and the behavioural intentions or necessary action to the perceiver [46]. In fact, Adenzato and colleagues [47] investigated the effect of tDCS over the medial frontal cortex on the theory of mind (ToM). They found that ToM performance in patients with PD-MCI was worse than that in healthy subjects, and ToM abilities were poorer in those with fronto-executive difficulties. These cues enable us to recognize another person’s emotional state and provide information on how to respond in these social situations.

Regarding behavioural tendencies, two opposite poles of human behaviour and motivation, approach and avoidance, are most pertinent. Gray’s theory posits two antipodal motivational systems, one appetitive (approach) and one aversive (avoidance), both forming the basis of human behaviour [48]. These systems are directly activated by perceived stimuli.

Happiness activates the hypothesized behavioural approach system since happy faces communicate an invitation to cooperate [49], whereas angry expressions, from the dominance-submission perspective, can lead to the establishment and maintenance of dominance hierarchies in social groups [50]. The evolutionary role of sadness, however, is more debated in the literature, as it cannot be linked directly to improved survival chances. Sadness has been proposed to stimulate caregiving and protective responses from others, and is therefore thought to play an important role in facilitating social bonding, reducing interpersonal aggression, and strengthening interpersonal relationships after a loss [51].

People with neurological or psychiatric disorders lose their ability to distinguish between pleasant and unpleasant experiences and the ability to assign the appropriate emotional valence to these experiences. This condition may lead to an inability to navigate the social environment in terms of interacting with others, understanding social context, and developing interpersonal relationships.

Over time, patients are unable to infer the emotional content from a situation or decide whether an experience is pleasurable or unpleasant. This leads to social withdrawal and isolation. Ferrucci and colleagues [52] found that tDCS applied for 20 min over the prefrontal cortex of depressed patients increased positive mood and alertness [52].

### Limitations

While our research revealed that PD patients become better at recognizing negative stimuli after tDCS, our study had several limitations. First, the sample size is very small for the generalization of the results; therefore, further studies are needed. Although we excluded psychiatric disorders through an interview, we did not evaluate anxiety or depression using a self-report questionnaire. This could affect activation by negative stimuli. Furthermore, we did not investigate how the changes in emotion recognition affect the daily lives of patients.

## Conclusion

Cerebellar stimulation could help to identify the neural mechanisms underlying FER and also help patients by enhancing FER, improving personal relationships, and reducing emotional disorders. Further studies involving bigger sample size and long follow-up periods should be conducted to investigate the duration of the effects of stimulation and the impact that therapy might have on the daily functional activities of patients.
